# Comparison of Two Artificial Intelligence Programs for Bone Age Assessment Using Hand–Wrist Radiographs in Korean Children and Adolescents Undergoing Orthodontic Diagnosis

**DOI:** 10.3390/children13091125

**Published:** 2026-08-22

**Authors:** Eun-ju Park, Hye-won Shin, So-youn An

**Affiliations:** 1 Department of Pediatric Dentistry, School of Dentistry, Chonnam National University, 33 Yongbong-ro, Buk-gu, Gwangju 61186, Republic of Korea; ejp92@naver.com; 2 Chaeumieum Dental Clinic, Ilsan-si 10544, Republic of Korea; nsa30725@naver.com; 3 Department of Pediatric Dentistry, College of Dentistry, Wonkwang University, Iksan-si 54538, Republic of Korea

**Keywords:** artificial intelligence, bone age assessment, skeletal maturation, hand–wrist radiograph, pediatric dentistry, orthodontics

## Abstract

**Purpose:** Accurate assessment of skeletal maturation is important for timing pediatric dental and orthodontic interventions. This study compared bone age estimates generated by two AI-based programs, CRESCOM MediAI-BA and VUNO Med-BoneAge^®^, in Korean children and adolescents undergoing orthodontic diagnostic evaluation. **Methods:** This retrospective study compared bone-age estimates generated by CRESCOM MediAI-BA and VUNO Med-BoneAge^®^ using left hand–wrist radiographs from 200 Korean children and adolescents (100 boys and 100 girls; 6–17 years) undergoing orthodontic diagnostic evaluation. Chronological age and program-derived bone age were compared by sex and age group using the Wilcoxon signed-rank test. Standardized differences were summarized using Cohen’s d, and Spearman correlations were calculated among chronological age, program-derived bone age, and the CRESCOM skeletal maturity indicator (SMI). **Results:** The standardized differences between chronological age and CRESCOM-predicted bone age were very small in boys (d = 0.143) and small in girls (d = 0.258). Corresponding differences for VUNO Med-BoneAge^®^ were small in boys (d = 0.254) and girls (d = 0.216). CRESCOM SMI correlated strongly with chronological age (ρ = 0.852), VUNO-predicted bone age (ρ = 0.910), and CRESCOM-predicted bone age (ρ = 0.932; all *p* < 0.001). However, statistically significant age- and sex-specific differences were observed between chronological age and AI-predicted bone age and between the two programs. **Conclusions:** CRESCOM MediAI-BA may provide clinically useful adjunctive information for skeletal-maturity assessment; nevertheless, AI-derived bone-age estimates from different programs should not be considered interchangeable and should be interpreted in conjunction with clinical findings and established skeletal-maturity indicators.

## 1. Introduction

In pediatric dentistry, treatment plans and their scope are determined by accurately assessing each patient’s growth status before treatment. However, since chronological age and physical development vary significantly among individuals, developmental age must be used to assess an individual’s physiological maturity. Various factors, such as skeletal maturity, dental maturity, somatic growth, and sexual maturity, are utilized to assess developmental age. Chronological age, which measures the time elapsed from birth, is used in growth studies and serves as the standard for all social and legal systems. However, evaluating an individual’s growth and developmental timing based on the average age for each growth stage is inappropriate due to high variability. Therefore, to accurately assess an individual’s growth and developmental timing, developmental age must be evaluated using chronological age as a baseline.

The standard clinical method for assessing bone maturity involves the use of radiographs of the cervical spine and the hand and wrist bones. Assessment of cervical spine maturity is based on standard lateral cephalometric radiographs taken as diagnostic data prior to orthodontic treatment [1,2]. Since the onset and extent of ossification, as well as the patterns of bone changes, vary among the individual bones of the hand and wrist as they mature, it is possible to infer an individual’s skeletal maturity [3]. Among the existing methods for assessing skeletal age using hand and wrist bones, the most representative are the Greulich–Pyle (GP) and Tanner–Whitehouse (TW) methods [4,5]. The GP method is highly subjective, relying on the similarity between images in the GP Atlas and the patient’s left wrist radiograph; it exhibits significant inter- and intra-observer variability, in addition to inter- and intra-institutional variability. Furthermore, there is no standardized protocol for bone assessment, and it is unclear which bones should be included in the evaluation [6,7]. With the advancement of deep learning—a subfield of artificial intelligence (AI) that utilizes artificial neural networks—several software programs have been developed to automate and standardize bone age assessment, thereby reducing inter-observer variability. BoneXpert is known as the first AI radiology system; it predicts bone age based on traditional machine learning methodologies, taking into account bone morphology, density, and the degree of epiphyseal fusion [8,9]. However, if the shape of a bone falls outside the range covered by the machine learning process, or if the bone age value deviates above a threshold compared to the average of all long bones, that bone is excluded from the calculation, and the final bone age is calculated using the evaluated bones. If fewer than eight bones are evaluated, the calculation may be inaccurate, so the X-ray is not evaluated; this is a major drawback of BoneXpert version 2.4.5.120. This method has been validated for bone age calculation in North American, Caucasian, African American, Hispanic, and Asian children with normal nutritional status, and has been reported to be applicable to various ethnic groups [10,11,12].

In orthodontic practice, skeletal maturation is commonly assessed using either cervical vertebral maturation (CVM) on lateral cephalometric radiographs or hand–wrist radiographs. CVM has the practical advantage that it can be assessed from lateral cephalometric radiographs routinely obtained for orthodontic diagnosis, thereby avoiding an additional radiograph. However, CVM assessment relies on morphological changes in a limited number of cervical vertebrae and may be influenced by image quality, head posture, vertebral superimposition, and observer-dependent interpretation. In contrast, hand–wrist radiographs provide information from multiple ossification centers, including the phalanges, metacarpals, carpal bones, and distal radius and ulna. This broader anatomical representation allows assessment of multiple developmental and fusion-related features across skeletal regions and forms the basis of established methods such as the Greulich–Pyle, Tanner–Whitehouse, and skeletal maturity indicator methods. Nevertheless, hand–wrist assessment requires a dedicated radiograph and should therefore be clinically justified. The ability of hand–wrist radiographs to capture a broad range of maturation features, together with the clinical relevance of SMI staging for orthodontic timing, provided the rationale for evaluating AI-based hand–wrist bone-age assessment systems in the present study [1,2,4,5,6,12].

VUNO Med-Bone Age^®^ is the first AI-based bone age assessment system approved by the Korean Ministry of Food and Drug Safety. This system was developed by analyzing 18,940 left wrist radiographs using the GP method [13,14]. The VUNO Korean bone age assessment method, based on deep learning, demonstrated superior performance compared to manual assessment using the GP Atlas. Compared to manual evaluation using the GP Atlas, the Korean model exhibits lower root mean square error and mean absolute error. VUNO provides the most probable estimated bone age based on the examined hand and wrist radiographs. In dental clinical practice, the skeletal maturity indicator (SMI) method, which classifies the ossification stages of six hand regions into 11 levels using four evaluation criteria, is the most commonly used method for assessing growth and development [15]. Additionally, the middle phalanx of the middle finger (MP3) method, which evaluates bone maturity in five stages by observing only the middle phalanx of the middle finger via periapical radiography, is also used for children and adolescents due to its simplicity and low radiation exposure [16]. MediAI-BA, introduced by Crescom, is a solution that uses artificial intelligence to automatically analyze hand and wrist X-rays of growing children and adolescents. It assists specialists in determining bone age and provides SMI (Skeletal Maturity Indicators) stages, which are crucial for determining the optimal timing for orthodontic treatment in children and adolescents. SMI is an indicator that analyzes the maturity of the hand and wrist bones to determine the appropriate timing for orthodontic treatment based on a child’s growth and developmental stage. Crescom MediAI-BA objectively provides SMI stages based on bone age, helping to identify growth stages—such as the growth spurt phase—in children and adolescents and supporting the determination of the optimal timing for orthodontic treatment. Previous studies have reported that AI-based evaluation methods demonstrate higher accuracy, reproducibility, and time efficiency compared to manual evaluation methods [7]. While BoneXpert versions 2.4.5.1 and 3.0.3 (Visiana, Hørsholm, Denmark) are among the most widely used methods, other AI-based bone age calculation software packages exist, including VUNO Med-Bone Age version 1.0.3 (VUNO) (VUNO, Seoul, Republic of Korea) (Table 1).

Despite the increasing clinical use of AI-based bone age assessment systems, direct comparisons between commercially available programs remain limited. In particular, to the best of our knowledge, no published study has compared VUNO Med-BoneAge^®^ and CRESCOM MediAI-BA in Korean children and adolescents undergoing orthodontic diagnostic evaluation. Therefore, this study aimed to compare bone age estimates generated by these two AI-based programs and to evaluate the association between the CRESCOM skeletal maturity indicator (SMI), chronological age, and AI-derived bone age estimates.

## 2. Materials and Methods

### 2.1. Selection and Eligibility Criteria

This retrospective study included patients aged 4–17 years who visited the Department of Pediatric Dentistry at Wonkwang University Daejeon Dental Hospital between January 2014 and December 2023 and underwent left hand–wrist radiography. A total of 560 patients (265 boys and 295 girls) were initially identified. The mean chronological age was 11.7 years for boys and 11.3 years for girls. Eligibility was restricted to patients with hand–wrist radiographs obtained using a Radio Multix UH X-ray system (Fujitsu-Siemens, Munich, Germany) under standardized exposure conditions (55 kVp and 4–5 mAs). Patients were excluded when a lateral cephalometric radiograph obtained within 2 months of the corresponding hand–wrist radiograph was unavailable. Furthermore, in the youngest and oldest age groups, only patients for whom both AI-based programs generated valid bone age estimates were included in the final analysis.

For the present analysis, left hand–wrist radiographs from 200 children and adolescents with no known conditions affecting skeletal development were selected. The final sample comprised 100 boys and 100 girls aged 6–17 years.

### 2.2. Ethical Considerations

The study protocol was approved by the Institutional Review Board of Wonkwang University (WKUIRB-202007-037; approved on 17 July 2020). The requirement for informed consent was waived because of the retrospective design of the study.

### 2.3. Hand–Wrist Radiography

Patients were seated with their left hand extended. The long axes of the hand and forearm were aligned parallel to the long axis of the detector. The third metacarpophalangeal joint was positioned at the center of the image field, and the X-ray beam was directed perpendicular to this joint. The distal radius and ulna were included in the radiographic field. Before AI analysis, radiographs were screened for adequate visualization of the distal radius and ulna, carpal bones, metacarpals, and phalanges, and for the absence of substantial motion artifact, positioning error, cropping, or exposure-related degradation that could interfere with bone-age assessment.

### 2.4. AI-Based Bone Age Assessment

Bone age was assessed using two AI-based programs: CRESCOM MediAI-BA (https://mediai.onzaram.com, 18 August 2026) and VUNO Med-BoneAge^®^ (https://boneage.maihub.ai, 18 August 2026). CRESCOM MediAI-BA provided both estimated bone age and an automated skeletal maturity indicator (SMI) stage. VUNO Med-BoneAge^®^ provided an estimated bone age based on analysis of hand–wrist radiographs. Both AI-based bone age assessment programs automatically identified the relevant anatomical landmarks and regions of interest on the hand–wrist radiographs and generated bone age estimates without manual annotation, verification, or correction by the evaluators.

### 2.5. Statistical Analysis

The Wilcoxon signed-rank test was used to compare paired values of chronological age and AI-predicted bone age and to compare estimated bone age values generated by VUNO Med-BoneAge^®^ and CRESCOM MediAI-BA. Analyses were performed separately by sex and age group.

Cohen’s d was calculated to assess the standardized difference between chronological age and estimated bone age generated by each AI program. Effect sizes were interpreted as very small (0.01 ≤ d < 0.20), small (0.20 ≤ d < 0.50), moderate (0.50 ≤ d < 0.80), large (0.80 ≤ d < 1.20), very large (1.20 ≤ d < 2.00), and huge (d ≥ 2.00).

Spearman rank correlation analysis was performed to evaluate the relationships among chronological age, VUNO-predicted bone age, CRESCOM-predicted bone age, and CRESCOM SMI. Statistical analyses were conducted using SPSS Statistics version 26.0 (IBM Corp., Armonk, NY, USA). A two-sided *p*-value < 0.05 was considered statistically significant.

## 3. Research Results

### 3.1. Standardized Differences Between Chronological Age and AI-Predicted Bone Age (Table 2)

#### 3.1.1. CRESCOM MediAI-BA

For CRESCOM MediAI-BA, Cohen’s d was 0.143 in boys and 0.258 in girls, indicating a very small standardized difference in boys and a small standardized difference in girls between chronological age and estimated bone age.

#### 3.1.2. VUNO Med-BoneAge^®^

For VUNO Med-BoneAge^®^, Cohen’s d was 0.254 in boys and 0.216 in girls, indicating small standardized differences between chronological age and estimated bone age in both sexes.

**Table 2:** Cohen’s d for the Differences between Chronological Age and AI-Predicted Bone Age.

Variables	Device Name (Class)	Cohen’s d	Interpretation
Chronological Age and CRESCOM Bone Age	Male	0.142933	Very small
	Female	0.258309	Small
Chronological Age and VUNOmed Bone Age	Male	0.254176	Small
	Female	0.216326	Small

Cohen’s d values are interpreted based on Cohen’s guidelines: 0.01 ≤ d < 0.2 = Very small; 0.2 ≤ d < 0.5 = Small; 0.5 ≤ d < 0.8 = Moderate; 0.8 ≤ d < 1.2 = Large; 1.2 ≤ d < 2.0 = Very large; and d ≥ 2.0 = Huge.

### 3.2. Comparison Between Chronological Age and VUNO Med-BoneAge^®^ Estimate

**Figure 1:**
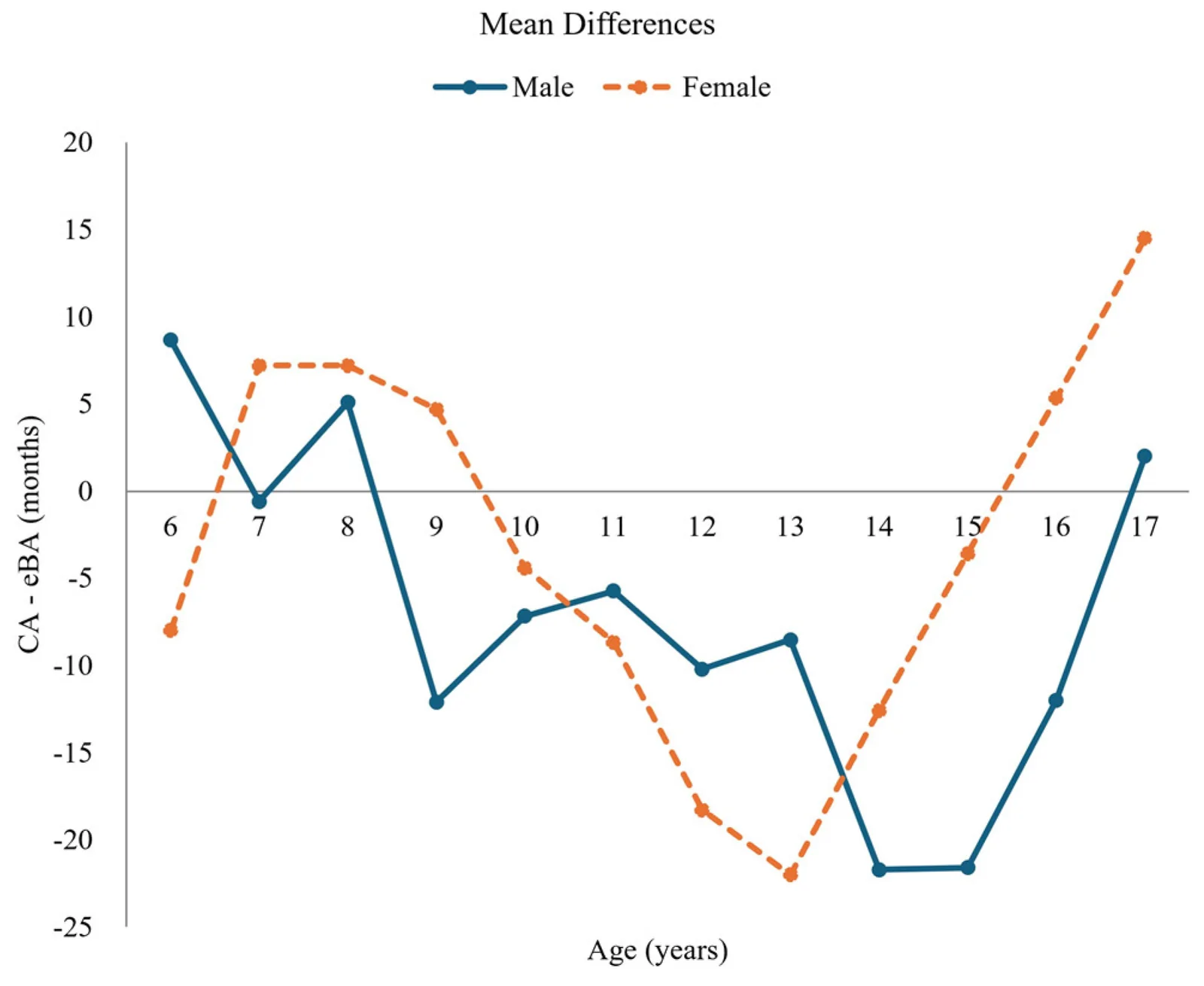
Mean differences between chronological age and estimated bone age generated by VUNO Med-BoneAge^®^, stratified by sex.

For boys, bone age assessment using VUNOmed-BoneAge^®^ showed that the estimated bone age was lower than the actual age at ages 6 and 8, but higher at age 7. From age 9 onward, the estimated bone age generally tended to be higher than the chronological age, with differences ranging from 5.73 to 21.71 months (Figure 1). Statistically significant differences were observed at ages 9, 10, 12, 13, 14, and 15 (*p* < 0.05).

For girls, the estimated bone age was lower than the chronological age at ages 7, 8, 9, 16, and 17, but higher at ages 6, 10, 11, 12, 13, 14, and 15 (Figure 1). The difference between estimated bone age and chronological age ranged from 3.57 to 22.00 months, with statistically significant differences observed at ages 11, 12, 13, and 14 (*p* < 0.05). Notably, at ages 12 and 13, the estimated bone age was 18.29 months and 22.00 months higher than the actual age, respectively, representing the largest differences within the female group.

To derive specific descriptive statistics by gender and age, and to verify whether there was a significant difference between actual age and bone age predicted using VUNOmed-BoneAge^®^, a Wilcoxon signed-rank test was conducted. The results are as follows (Table 3, Figure 1).

### 3.3. Comparison Between Chronological Age and CRESCOM MediAI-BA Estimates

For boys, the estimated bone age was lower than the actual age between 6 and 8 years old; at age 8, the estimated bone age was significantly lower than the actual age by 9.40 (*p* = 0.033). From age 9 onward, with the exception of age 17, estimated bone age generally tended to be higher than chronological age, with differences ranging from 2.00 to 20.29 months. In particular, statistically significant differences were observed at ages 13, 14, and 15 (*p* < 0.05), with the largest difference observed at age 14 (20.29).

For girls, with the exception of ages 7 and 16–17, estimated bone age tended to be higher than chronological age, with the difference ranging from 1.20 to 17.71 months. In particular, statistically significant differences were observed in the 11-, 12-, 13-, and 14-year-old groups, where estimated bone age was significantly higher than chronological age (*p* < 0.05); the largest differences were observed in the 12-year-old group (15.21) and the 13-year-old group (17.71).

**Figure 2:**
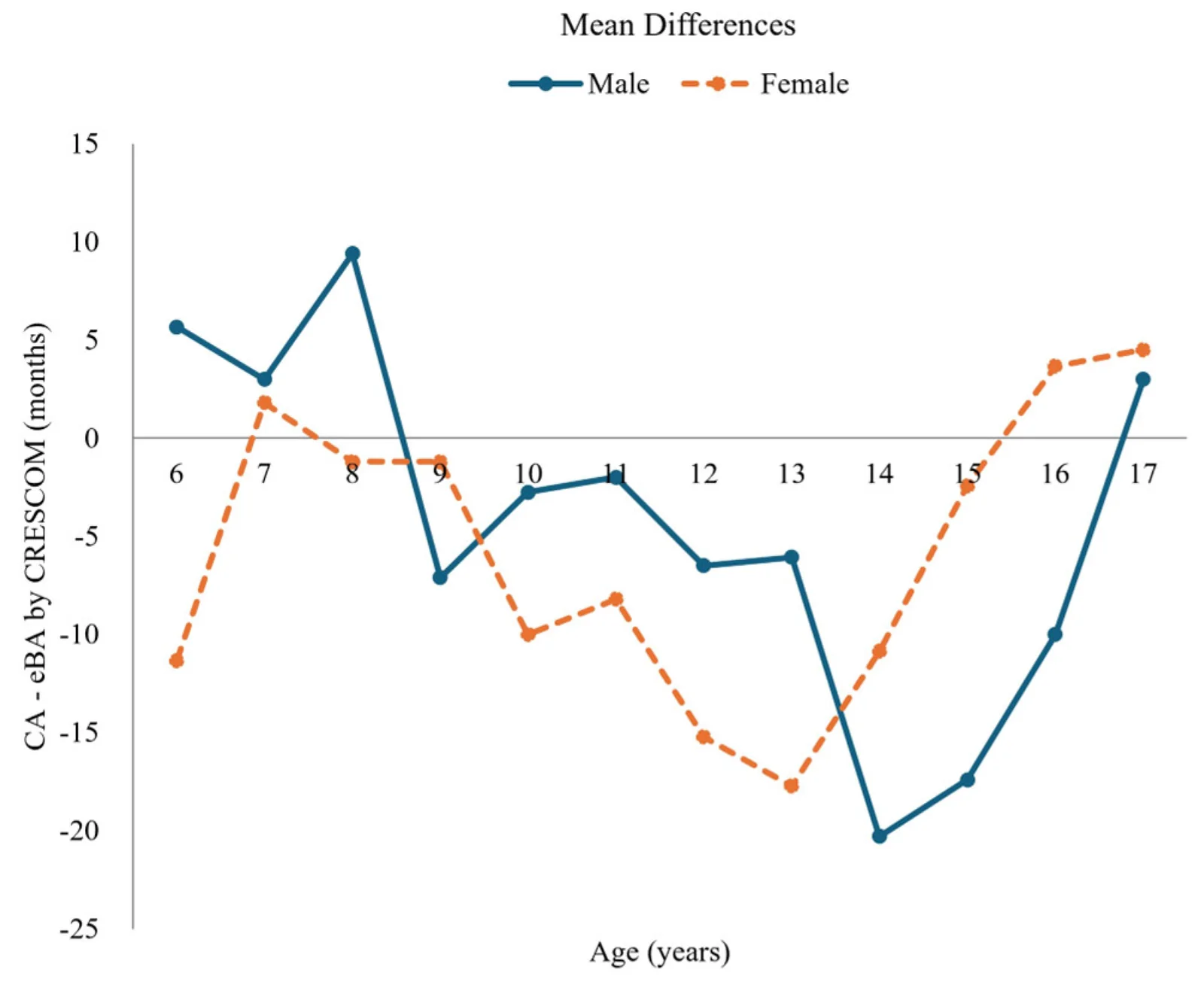
Mean differences between chronological age and estimated bone age generated by CRESCOM MediAI-BA, stratified by sex.

To derive specific descriptive statistics by gender and age and to verify whether there was a significant difference between actual age and the predicted bone age using CRESCOM Mediai-BA, a Wilcoxon signed-rank test was conducted, and the results are as follows (Table 4, Figure 2).

### 3.4. Comparison of Estimated Bone Age Between VUNO Med-BoneAge^®^ and CRESCOM MediAI-BA

For boys, CRESCOM-predicted bone age was higher than VUNOmed-predicted bone age only at age 6; from age 7 onward, CRESCOM-predicted bone age was consistently lower than VUNOmed-predicted bone age, with the difference ranging from 1.00 to 5.00 months. In particular, for ages 9, 10, 11, 12, and 13, the CRESCOM-predicted bone age was found to be significantly lower than the VUNOmed-predicted bone age (*p* < 0.05), with the largest difference observed at age 9 (5.00).

For girls, the CRESCOM-predicted skeletal age was higher than the VUNOmed-predicted skeletal age at ages 6, 7, 8, 9, 10, 16, and 17, with the difference ranging from 3.33 to 10.00 months. In contrast, at ages 11, 12, 13, 14, and 15, the CRESCOM-predicted bone age was relatively lower than the VUNOmed-predicted bone age had a difference ranging from 0.47 to 4.29 months. In particular, it was verified that the CRESCOM-predicted skeletal age was significantly higher than the VUNOmed-predicted skeletal age for 7-, 8-, 9-, 14-, and 15-year-olds (*p* < 0.05), with the largest difference observed at age 8 (8.40). Conversely, it was verified that the CRESCOM-predicted skeletal age was significantly lower than the VUNOmed-predicted skeletal age for 13-year-olds (*p* < 0.05).

**Figure 3:**
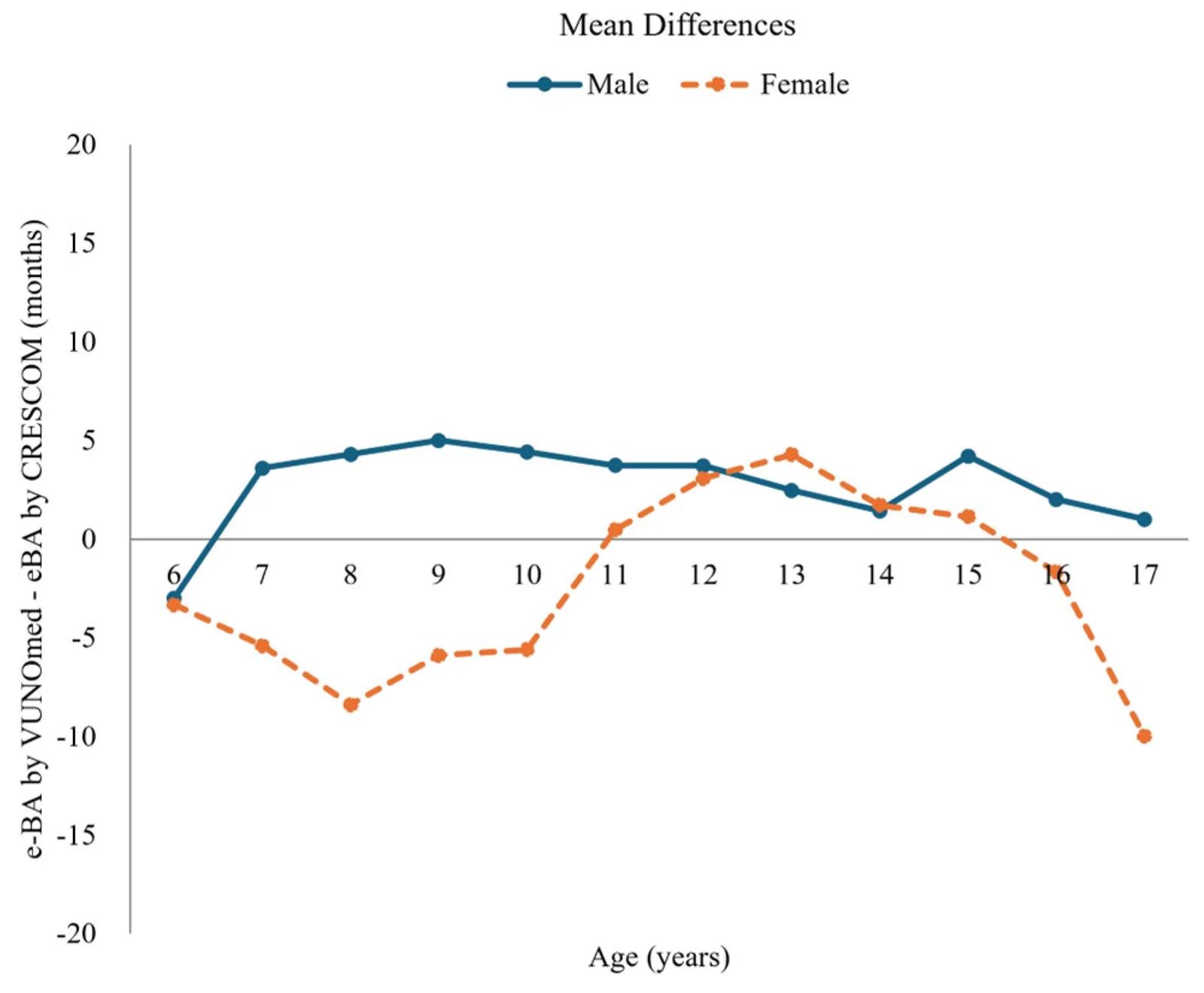
Mean differences in estimated bone age between VUNO Med-BoneAge^®^ and CRESCOM MediAI-BA, stratified by sex.

To verify whether there were significant differences between the bone ages predicted by VUNOmed and those predicted by CRESCOM MediAI-BA by gender and age, a Wilcoxon signed-rank test was conducted, and the results are as follows (Table 5, Figure 3).

### 3.5. Correlations Among Chronological Age, AI-Predicted Bone Age, and CRESCOM SMI

Spearman correlation analysis demonstrated statistically significant, strong positive correlations among chronological age, VUNO-predicted bone age, CRESCOM-predicted bone age, and CRESCOM SMI in the total sample and in both sex-specific groups (all *p* < 0.001). In the overall sample, CRESCOM SMI was strongly correlated with chronological age (ρ = 0.852), VUNO-predicted bone age (ρ = 0.910), and CRESCOM-predicted bone age (ρ = 0.932).

To determine the correlations between actual age, bone age estimated by VUNOmed, bone age estimated by CRESCOM, and CRESCOM SMI, we performed Spearman’s correlation analysis, a nonparametric correlation method, and the results are as follows (Table 6).

## 4. Discussion

The present study compared the bone age estimates generated by two artificial intelligence (AI)-based programs, CRESCOM MediAI-BA and VUNO Med-BoneAge^®^, in Korean children and adolescents undergoing orthodontic diagnostic evaluation. Overall, CRESCOM-predicted bone age demonstrated a small discrepancy from chronological age, with Cohen’s d values of 0.143 for boys and 0.258 for girls. In addition, the CRESCOM skeletal maturity indicator (SMI) showed very strong positive correlations with chronological age, VUNO-predicted bone age, and CRESCOM-predicted bone age. These findings suggest that CRESCOM MediAI-BA may provide clinically useful information regarding skeletal maturation and growth status in pediatric dental patients. However, the results also showed age- and sex-specific differences between chronological age and AI-predicted bone age, as well as between the two AI programs, indicating that the outputs of these systems should not be interpreted as interchangeable. Accurate assessment of skeletal maturity is particularly important in pediatric dentistry and orthodontics because the timing of several orthopedic and orthodontic interventions is closely related to the pubertal growth spurt. BAA does not introduce a new technical field in dentistry. This is because, during the Roman Empire, the eruption of the second molar was already used as an indicator for conscripting young men into military service [17]. In the 19th century, dentists estimated age, considering tooth eruption a reliable method for determining a child’s age. At that time in Britain, the minimum skeletal age was calculated to be 7 years [18]. Although physical growth and facial growth are correlated, it is difficult to use this relationship to reliably predict changes in facial growth or to apply it with clinical accuracy. A cautious approach is necessary when using these factors to assess growth.

Methods for assessing bone age include peak height velocity (PHV) and hand–wrist radiographs. According to a survey by the Korean Society of Pediatrics, Korean girls experience their maximum height increase at an average age of 12.3 years, with an average height of approximately 146 cm at that time. Generally, girls’ maximum height growth period occurs about one year after the onset of breast development or about one year before menarche. The average maximum height gain for girls over a one-year period is approximately 8 cm (ranging from 6–11 cm). The average age of menarche for girls is 12.8 years. In contrast, boys experience their maximum height growth at an average age of 13.9 years, with an average height of approximately 159 cm at that time. Puberty in boys begins later than in girls, typically around age 12 (ranging from 9–14 years). Boys achieve 84% of their final height around age 12 and reach 95% of their final height around age 15. Generally, the total height gain during the 5–6 years of puberty in boys is about 28 cm, with the maximum annual height gain averaging about 10 cm (ranging from 7–12 cm) [19].

Hand–wrist radiographs have traditionally been used to evaluate skeletal maturation through methods such as the Greulich–Pyle (GP), Tanner–Whitehouse (TW), and skeletal maturity indicator methods [5,6,16]. Nevertheless, conventional visual assessment depends substantially on examiner experience and may be affected by inter- and intra-observer variability [7,8]. In this context, AI-based bone age assessment has the potential to improve objectivity, reproducibility, and efficiency of skeletal maturity evaluation. The current findings are consistent with previous reports indicating that AI-assisted bone age assessment can reduce observer-dependent variation and provide rapid, standardized estimates of bone age [8]. The relatively small effect sizes observed between chronological age and CRESCOM-predicted bone age indicate that, at the overall group level, the discrepancy between the two measures was limited. This finding supports the potential applicability of CRESCOM MediAI-BA as an adjunctive tool for bone age assessment in Korean children and adolescents. However, the direction and magnitude of the difference varied according to age and sex. In boys, CRESCOM-predicted bone age tended to be lower than chronological age at younger ages but higher than chronological age from 9 years onward, except at 17 years. The largest difference was observed among 14-year-old boys, in whom the estimated bone age exceeded chronological age by approximately 20 months. In girls, CRESCOM-predicted bone age was generally higher than chronological age, particularly in the 11- to 14-year age groups. These age ranges correspond broadly to the period of accelerated pubertal growth and rapid skeletal maturation. Therefore, the greater discrepancy observed during these periods may reflect the substantial biological variability in the timing and velocity of maturation among individuals rather than a uniform limitation of the AI system. Sex-related differences should also be interpreted in light of the distinct maturational patterns of boys and girls. Girls generally experience the pubertal growth spurt earlier than boys, and skeletal maturation may progress rapidly during the early adolescent period. In the present study, the greatest overestimation by CRESCOM was observed in 12- and 13-year-old girls, whereas the largest difference in boys occurred at 14 years. These findings appear to correspond with the expected sex-specific timing of pubertal maturation. However, because chronological age is not a direct reference standard for skeletal maturity, the difference between chronological age and estimated bone age does not necessarily indicate an error by the AI program. Rather, it may represent a true advancement or delay in biological maturation relative to calendar age. Future studies incorporating expert-derived GP, TW, or SMI assessments, as well as longitudinal growth data, are needed to determine whether the observed differences represent systematic bias or individual variation in skeletal maturation. Although both AI programs showed strong correlations with chronological age, their estimated bone ages were not completely consistent across all age groups. In boys, CRESCOM-predicted bone age was generally lower than VUNO Med-BoneAge^®^ from 7 years onward, with significant differences observed between 9 and 13 years of age. In girls, the direction of the difference varied across age groups: CRESCOM-predicted bone age was higher than that predicted by VUNO in younger children and at 16–17 years of age, whereas it was lower during much of the adolescent period. These findings suggest that the two programs may be influenced by differences in their training datasets, algorithmic architecture, reference standards, image preprocessing procedures, or approaches to handling specific skeletal maturation features. VUNO Med-BoneAge^®^ was developed using a large number of Korean hand–wrist radiographs interpreted according to the GP method [14,15], whereas CRESCOM MediAI-BA was designed not only to estimate bone age but also to provide an SMI stage relevant to orthodontic growth evaluation. Accordingly, the outputs of the two programs may reflect different underlying calibration methods and clinical objectives. The strong correlation between CRESCOM SMI and both chronological age and AI-estimated bone age is clinically noteworthy. In particular, the correlation between CRESCOM SMI and CRESCOM-predicted bone age was very high in the overall sample and in both sexes. This result indicates that the SMI output of CRESCOM changes consistently with increasing skeletal maturity. Because SMI staging has been widely used in orthodontics to identify developmental stages and estimate the timing of the pubertal growth spurt [16], automated SMI provision may be especially valuable in pediatric dental practice. An AI-based system that simultaneously provides bone age and skeletal maturity stage could support clinicians in evaluating growth potential and planning growth-modification treatment. Nevertheless, the high correlation observed in this study does not establish direct equivalence between CRESCOM SMI and manual SMI assessment. Further validation against conventional SMI staging by experienced examiners is required before CRESCOM SMI can be considered a substitute for established manual methods. This study has several limitations. First, the retrospective design and single-center setting may limit the generalizability of the results to other populations and clinical environments. Second, although the sample was balanced by sex, the number of participants in each individual age group was limited; therefore, age-specific findings should be interpreted cautiously. Third, chronological age was used as a comparison measure, but it is not a gold standard for skeletal maturity because substantial individual variation exists in biological development. A more rigorous validation should compare AI-derived bone age and SMI results with assessments performed by multiple experienced observers using established methods, such as the GP, TW, and manual SMI methods. Fourth, the present study evaluated the agreement and correlations between AI outputs but did not assess intra-program repeatability, image-quality-related failure rates, or the clinical consequences of discrepant results for orthodontic treatment timing. Finally, the cross-sectional design did not allow evaluation of whether AI-derived bone age or SMI more accurately predicts future pubertal growth or treatment response. Despite these limitations, this study is meaningful because it provides an initial comparison of two AI-based bone age assessment programs used in Korean children and adolescents. CRESCOM MediAI-BA showed a relatively small overall difference from chronological age and demonstrated strong correlations among its bone age and SMI outputs and the VUNO Med-BoneAge^®^ estimates. However, significant age- and sex-dependent differences were observed between CRESCOM and VUNO Med-BoneAge^®^, particularly during periods of rapid skeletal maturation. Therefore, clinicians should interpret AI-generated bone age values in conjunction with clinical findings, radiographic features, growth history, and other skeletal maturity indicators rather than relying on a single numerical output. Larger multicenter longitudinal studies are needed to validate the accuracy of CRESCOM MediAI-BA against established reference standards and to determine its value in predicting the timing of pubertal growth and optimizing orthodontic treatment planning.

This study has several limitations. First, the retrospective single-center design and the predominance of participants from the Daejeon and Chungcheong regions may limit the generalizability of the findings. Second, although the overall sample was balanced by sex, the number of participants in individual age groups was limited, precluding more detailed analyses by half-year intervals. Third, children younger than 5 years were not included. Fourth, chronological age was used as a comparator but is not a gold standard for skeletal maturity because biological development varies substantially among individuals. Future studies should compare AI-derived bone age and SMI values with assessments performed independently by experienced examiners using established GP, TW, and manual SMI methods. Finally, longitudinal multicenter studies are needed to determine whether AI-derived bone age and SMI improve prediction of pubertal growth and orthodontic treatment response.

## 5. Conclusions

CRESCOM MediAI-BA demonstrated a small overall standardized difference from chronological age in Korean children and adolescents undergoing orthodontic diagnostic evaluation. Its automated skeletal maturity indicator showed strong correlations with chronological age and with bone age estimates generated by both CRESCOM MediAI-BA and VUNO Med-BoneAge^®^. However, the two AI programs demonstrated age- and sex-dependent differences, particularly during periods of rapid skeletal maturation. Therefore, AI-derived bone age estimates should not be considered interchangeable and should be interpreted together with clinical findings, radiographic characteristics, growth history, and established skeletal maturity indicators. Further multicenter longitudinal validation against expert-derived reference standards is warranted.

## Figures and Tables

**Table 1:** Overview of Approved and Certified Artificial Intelligence Medical Devices in Korea.

Company Name	Device Name (Class)	Approval No. (Approval Date)	Intended Use	Product Appearance
VUNO Co., Ltd. (Seoul, Republic of Korea)	Medical Image Analysis Device Software (Class II)	Approval No. 18–360 (16 May 2018)	Bone Age Assessment Software	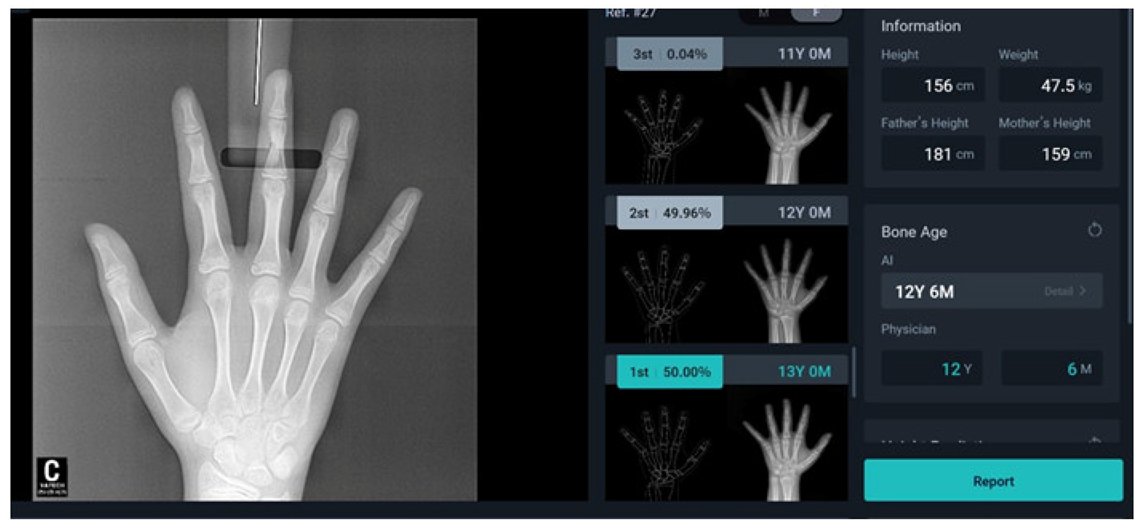
Crescom Co., Ltd. (Seongnam-si, Republic of Korea)	Medical Image Analysis Device Software (Class II)	Approval No. 20–153 (3 March 2020)	Bone Age Assessment Software	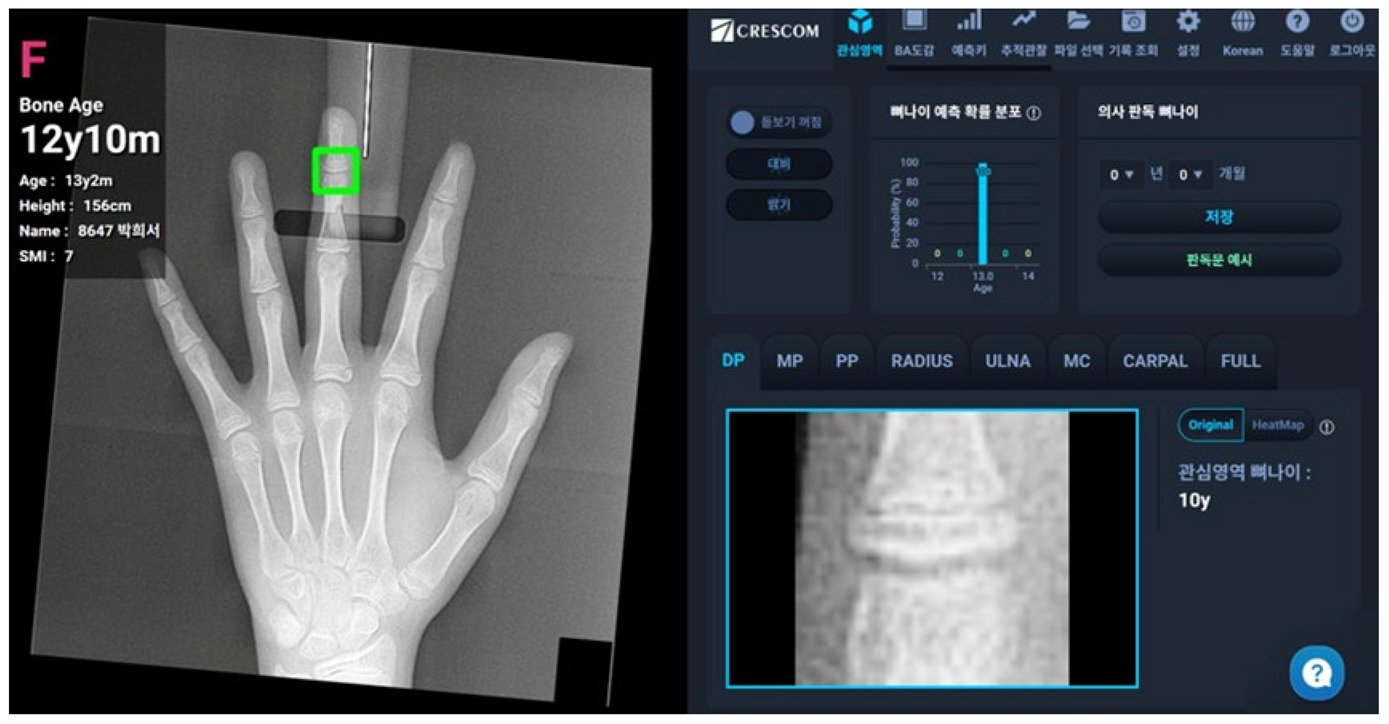
HealthHub Co., Ltd. (Seoul, Republic of Korea)	Medical Image Analysis Device Software (Class II)	Approval No. 20–166 (10 March 2020)	Bone Age Assessment Software	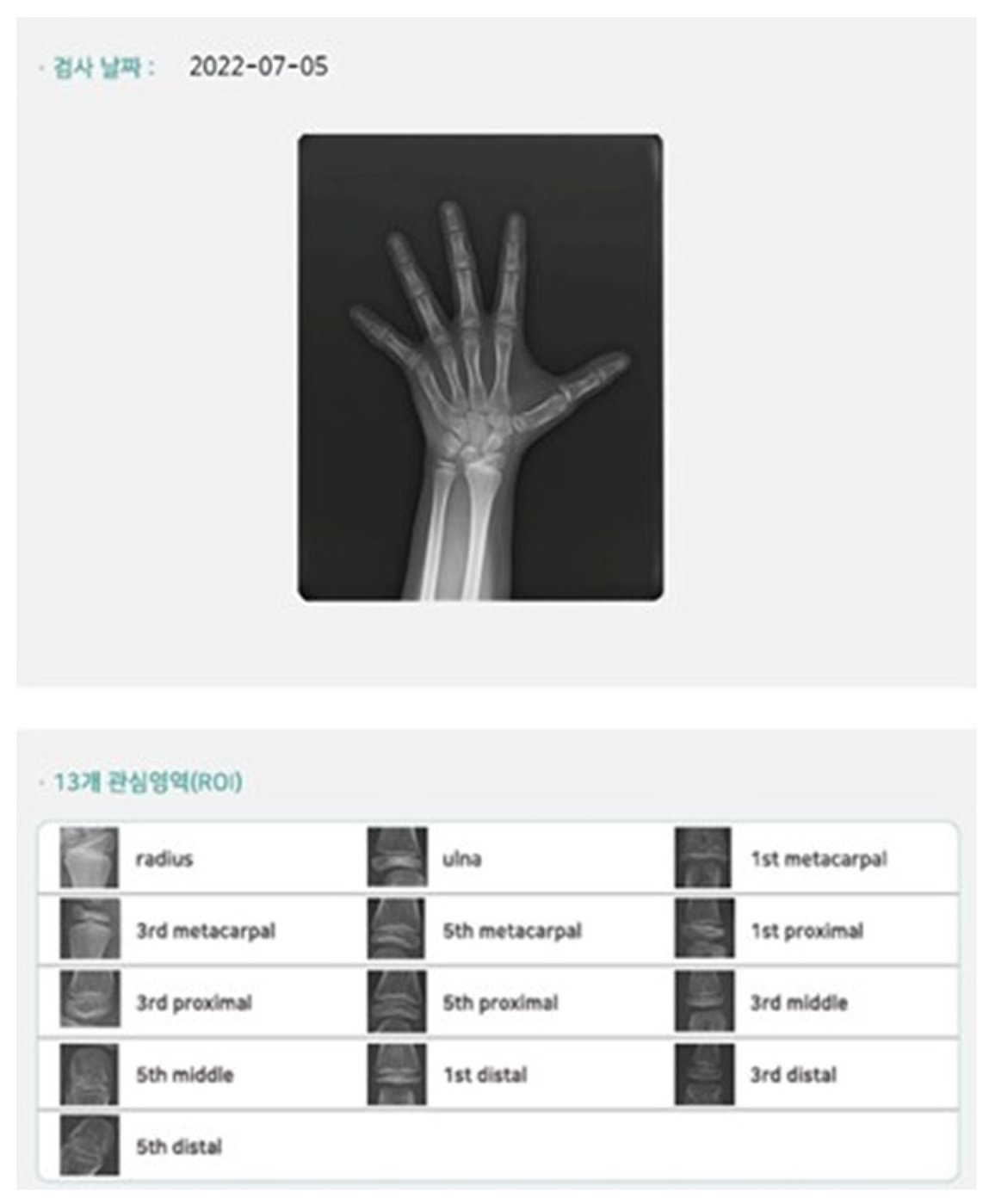

**Table 3:** Differences between Chronological Age and Estimated Bone Age Generated by VUNO Med-BoneAge^®^, Stratified by Sex and Age.

Sex	Age	N	CA (Months)		e-BA (Months)		CA-e-BA		*p*-Value
			Mean	SD	Mean	SD	Mean	SD	
Male	6	3	78.33	4.04	69.67	12.70	8.67	16.07	0.593
	7	5	91.80	4.09	92.40	15.04	−0.60	15.82	0.893
	8	10	100.20	3.12	95.10	15.47	5.10	14.80	0.285
	9	10	115.20	4.02	127.30	14.77	−12.10	12.33	**0.019**
	10	12	123.75	2.90	130.92	8.56	−7.17	8.13	**0.015**
	11	15	137.07	3.58	142.80	10.98	−5.73	12.43	0.096
	12	14	149.71	3.97	159.93	13.37	−10.21	13.51	**0.026**
	13	15	162.07	3.06	170.60	17.67	−8.53	15.93	**0.017**
	14	7	172.71	3.99	194.43	17.10	−21.71	15.23	**0.028**
	15	5	182.40	2.07	204.00	6.28	−21.60	6.27	**0.043**
	16	2	197.00	1.41	209.00	2.83	−12.00	4.24	0.180
	17	2	211.00	4.24	209.00	4.24	2.00	0.00	0.157
Female	6	3	75.67	4.62	83.67	7.64	−8.00	3.61	0.109
	7	5	90.80	3.96	83.60	6.27	7.20	5.72	0.066
	8	10	102.90	3.54	95.70	13.38	7.20	11.66	0.059
	9	10	113.60	3.57	108.90	19.35	4.70	18.50	0.444
	10	10	125.40	3.17	129.80	15.10	−4.40	14.08	0.333
	11	15	138.07	3.17	146.73	14.99	−8.67	14.79	**0.048**
	12	14	151.21	3.42	169.50	13.10	−18.29	11.26	**0.002**
	13	14	161.07	3.73	183.07	12.42	−22.00	10.33	**0.001**
	14	7	173.14	3.98	185.71	9.98	−12.57	8.14	**0.028**
	15	7	184.29	2.56	187.86	5.43	−3.57	5.47	0.108
	16	3	196.33	3.79	191.00	2.65	5.33	1.15	0.102
	17	2	207.00	4.24	192.50	2.12	14.50	6.36	0.180

CA, chronological age; e-BA, estimated bone age; SD, standard deviation.

**Table 4:** Differences between Chronological Age and Estimated Bone Age Generated by CRESCOM MediAI-BA, Stratified by Sex and Age.

Sex	Age	N	CA (Months)		e-BA (Months)		CA-e-BA		*p*-Value
			Mean	SD	Mean	SD	Mean	SD	
Male	6	3	78.33	4.04	72.67	8.08	5.67	10.69	0.655
	7	5	91.80	4.09	88.80	13.86	3.00	14.88	0.345
	8	10	100.20	3.12	90.80	11.01	9.40	11.39	**0.033**
	9	10	115.20	4.02	122.30	16.36	−7.10	13.82	0.139
	10	12	123.75	2.90	126.50	9.62	−2.75	9.72	0.504
	11	15	137.07	3.58	139.07	12.26	−2.00	13.60	0.362
	12	14	149.71	3.97	156.21	12.67	−6.50	12.91	0.084
	13	15	162.07	3.06	168.13	16.59	−6.07	14.74	**0.022**
	14	7	172.71	3.99	193.00	14.48	−20.29	12.28	**0.028**
	15	5	182.40	2.07	199.80	9.04	−17.40	8.65	**0.043**
	16	2	197.00	1.41	207.00	8.49	−10.00	9.90	0.180
	17	2	211.00	4.24	208.00	8.49	3.00	12.73	0.655
Female	6	3	75.67	4.62	87.00	10.58	−11.33	6.11	0.109
	7	5	90.80	3.96	89.00	7.71	1.80	6.80	0.465
	8	10	102.90	3.54	104.10	14.75	−1.20	12.45	0.329
	9	10	113.60	3.57	114.80	19.48	−1.20	18.78	0.635
	10	10	125.40	3.17	135.40	14.64	−10.00	14.56	0.092
	11	15	138.07	3.17	146.27	12.97	−8.20	12.98	**0.030**
	12	14	151.21	3.42	166.43	11.62	−15.21	9.69	**0.001**
	13	14	161.07	3.73	178.79	13.11	−17.71	11.32	**0.001**
	14	7	173.14	3.98	184.00	12.17	−10.86	10.67	**0.043**
	15	7	184.29	2.56	186.71	11.03	−2.43	10.86	0.446
	16	3	196.33	3.79	192.67	10.21	3.67	6.43	0.414
	17	2	207.00	4.24	202.50	4.95	4.50	9.19	0.655

CA, chronological age; e-BA, estimated bone age; SD, standard deviation.

**Table 5:** Differences in Estimated Bone Age between VUNO Med-BoneAge^®^ and CRESCOM MediAI-BA, Stratified by Sex and Age.

Sex	Age	N	VUNOmed (Months)		CRESCOM (Months)		VUNOmed-CRESCOM		*p*-Value
			Mean	SD	Mean	SD	Mean	SD	
Male	6	3	69.67	12.70	72.67	8.08	−3.00	6.00	0.414
	7	5	92.40	15.04	88.80	13.86	3.60	5.13	0.102
	8	10	95.10	15.47	90.80	11.01	4.30	7.93	0.081
	9	10	127.30	14.77	122.30	16.36	5.00	3.46	**0.007**
	10	12	130.92	8.56	126.50	9.62	4.42	4.54	**0.007**
	11	15	142.80	10.98	139.07	12.26	3.73	5.46	**0.024**
	12	14	159.93	13.37	156.21	12.67	3.71	4.41	**0.010**
	13	15	170.60	17.67	168.13	16.59	2.47	2.92	**0.009**
	14	7	194.43	17.10	193.00	14.48	1.43	4.35	0.497
	15	5	204.00	6.28	199.80	9.04	4.20	4.44	0.104
	16	2	209.00	2.83	207.00	8.49	2.00	5.66	0.655
	17	2	209.00	4.24	208.00	8.49	1.00	12.73	0.655
Female	6	3	83.67	7.64	87.00	10.58	−3.33	3.21	0.109
	7	5	83.60	6.27	89.00	7.71	−5.40	1.67	**0.042**
	8	10	95.70	13.38	104.10	14.75	−8.40	4.40	**0.005**
	9	10	108.90	19.35	114.80	19.48	−5.90	3.28	**0.005**
	10	10	129.80	15.10	135.40	14.64	−5.60	9.01	0.068
	11	15	146.73	14.99	146.27	12.97	0.47	5.29	0.488
	12	14	169.50	13.10	166.43	11.62	3.07	5.86	0.091
	13	14	183.07	12.42	178.79	13.11	4.29	6.72	**0.027**
	14	7	185.71	9.98	184.00	12.17	1.71	6.24	0.344
	15	7	187.86	5.43	186.71	11.03	1.14	6.26	0.672
	16	3	191.00	2.65	192.67	10.21	−1.67	7.57	0.785
	17	2	192.50	2.12	202.50	4.95	−10.00	2.83	0.180

**Table 6:** Spearman Correlations among Chronological Age, AI-Predicted Bone Age, and CRESCOM Skeletal Maturity Indicator.

Group	Variables	1	2	3	4
		R (p)	R (p)	R (p)	R (p)
Total	1. Chronological age	1			
	2. e-BA by VUNOmed	0.939 (<0.001)	1		
	3. e-BA by CRESCOM	0.938(<0.001)	0.989 (<0.001)	1	
	4. CRESCOM SMI	0.852 (<0.001)	0.910 (<0.001)	0.932 (<0.001)	1
Male	1. Chronological age	1			
	2. e-BA by VUNOmed	0.930 (<0.001)	1		
	3. e-BA by CRESCOM	0.935 (<0.001)	0.992 (<0.001)	1	
	4. CRESCOM SMI	0.881 (<0.001)	0.962 (<0.001)	0.964 (<0.001)	1
Female	1. Chronological age	1			
	2. e-BA by VUNOmed	0.947 (<0.001)	1		
	3. e-BA by CRESCOM	0.941 (<0.001)	0.990 (<0.001)	1	
	4. CRESCOM SMI	0.927 (<0.001)	0.970 (<0.001)	0.982 (<0.001)	1

## Data Availability

The datasets used and/or analyzed during the current study are available from the corresponding author on reasonable request.
